# Exogenous application of zinc (Zn) at the heading stage regulates 2-acetyl-1-pyrroline (2-AP) biosynthesis in different fragrant rice genotypes

**DOI:** 10.1038/s41598-019-56159-7

**Published:** 2019-12-20

**Authors:** Haowen Luo, Bin Du, Longxin He, Jing He, Lian Hu, Shenggang Pan, Xiangru Tang

**Affiliations:** 1 Department of Crop Science and Technology, College of Agriculture, South China Agricultural University, Guangzhou, 510642 P.R. China; 2 Scientific Observation and Experimental Station of Crop Cultivation in South China, Ministry of Agriculture, Guangzhou, 510642 P.R. China; 3 College of Engineering, South China Agricultural University, Guangzhou, 510642 P.R. China

**Keywords:** Enzymes, Photosynthesis, Plant development, Plant physiology

## Abstract

Zinc (Zn) is an important microelement for rice and plays a key role in many physiological processes. This study assessed the physio-biochemical responses involved in biosynthesis of 2-acety-1-pyrroline (2-AP), which is a key compound in the aroma of fragrant rice, in four different fragrant rice varieties, i.e., *Meixiangzhan-2*, *Xiangyaxiangzhan*, *Ruanhuayou-134*, and *Yunjingyou*. Four concentrations (0, 0.50, 1.00 and 2.00 g L^−1^) of zinc chloride were applied to fragrant rice foliage at the heading stage and named CK, Zn1, Zn2 and Zn3, respectively. Our results showed that compared with CK, the Zn1, Zn2 and Zn3 treatments all significantly increased the 2-AP concentration in mature grains of the four fragrant rice genotypes. Furthermore, exogenous application of Zn not only enhanced the activities of enzymes, including proline dehydrogenase (PDH), △1-pyrroline-5-carboxylic acid synthetase (P5CS), and diamine oxidase (DAO), which are involved in 2-AP biosynthesis, but also improved the contents of the related precursors, such as Δ1-pyrroline, proline and pyrroline-5-carboxylic acid (P5C). In addition, compared to the CK treatment, the Zn2 treatment markedly increased the net photosynthetic rate of fragrant rice during the grain filling stage and increased the seed-setting rate, 1000-grain weight and grain yield in all fragrant rice genotypes. Foliar application of Zn also markedly increased the grain Zn content. In general, 1.00 g L^−1^ seemed to be the most suitable application concentration because the highest 2-AP content and grain weight were recorded with this treatment.

## Introduction

Fragrant rice is a special type of rice that carries a special aroma. In recent years, the market price of fragrant rice has greatly increased^1^. Therefore, many researchers and farmers have begun to pay attention to fragrant rice production and quality regulation. As the most special characteristic of fragrant rice, the aroma is a determining factor in fragrant rice quality and significantly affects the price of fragrant rice^2^. It is generally believed that 2-acetyl-1-pyrroline (2-AP) is the primary component responsible for the fragrant rice aroma^3^, and a few studies have shown that the exogenous application of some metallic and non-metallic elements could markedly improve the 2-AP content in fragrant rice. For example, the study by Li *et al*.^4^ illustrated that application of manganese (Mn) induced regulation of 2-AP biosynthesis in fragrant rice. A previous study demonstrated that the application of silicon (Si) fertilizer could improve both the photosynthetic rate and 2-AP content of fragrant rice^5^. Thus, exogenous application of microelements could be an effective way to regulate 2-AP formation in fragrant rice.

Zinc (Zn) is an essential micronutrient for the normal growth and development of crops, and it plays an important physiological and biochemical role^6^. Zn is also an essential micronutrient in human health. Zn deficiency ranks 11th among more than 20 factors causing disease worldwide and 5th among the top 10 factors causing disease in developing countries^7^. Zn deficiency often leads to physical decline, vision loss or blindness, mental retardation, reduced immune function and disease resistance, and a high mortality and disease rate^8^. The study by Fu *et al*.^9^ also revealed that the reduction in photosynthesis under Zn deficiency was primarily due to non-stomatal limitations, in particular the changes in photosystem II (PS II). Therefore, in recent years, the nutritional status of plant trace elements and their relationship with human health, especially the biological fortification of iron (Fe) and Zn in cereal crops, has attracted increasing attention from researchers. Han *et al*.^10^ indicated that foliar application of Zn increased the leaf Zn concentration, thereby maintaining superoxide dismutase activity and membrane stability and protecting the photosynthetic apparatus against heat damage. Mo *et al*.^11^ revealed that supplementation with Zn could improve the 2-AP content in detached fragrant rice panicles *in vitro*. However, there have been no reports about the effect of foliar application of Zn on fragrant rice performance in field crop production, and the mechanism by which Zn application affects 2-AP biosynthesis in fragrant rice is still unclear.

Hence, the objective of this study was to evaluate 2-AP biosynthesis under foliar application of Zn at different concentrations using four fragrant rice varieties. The activities of five enzymes and contents of three precursors and two related compounds involved in 2-AP biosynthesis in fragrant rice were investigated. The grain yield and related trials were also estimated. We hope that this study will be useful for increasing the aroma in cultivated fragrant rice.

## Results

### 2-AP concentration in mature grains

**Figure 1:**
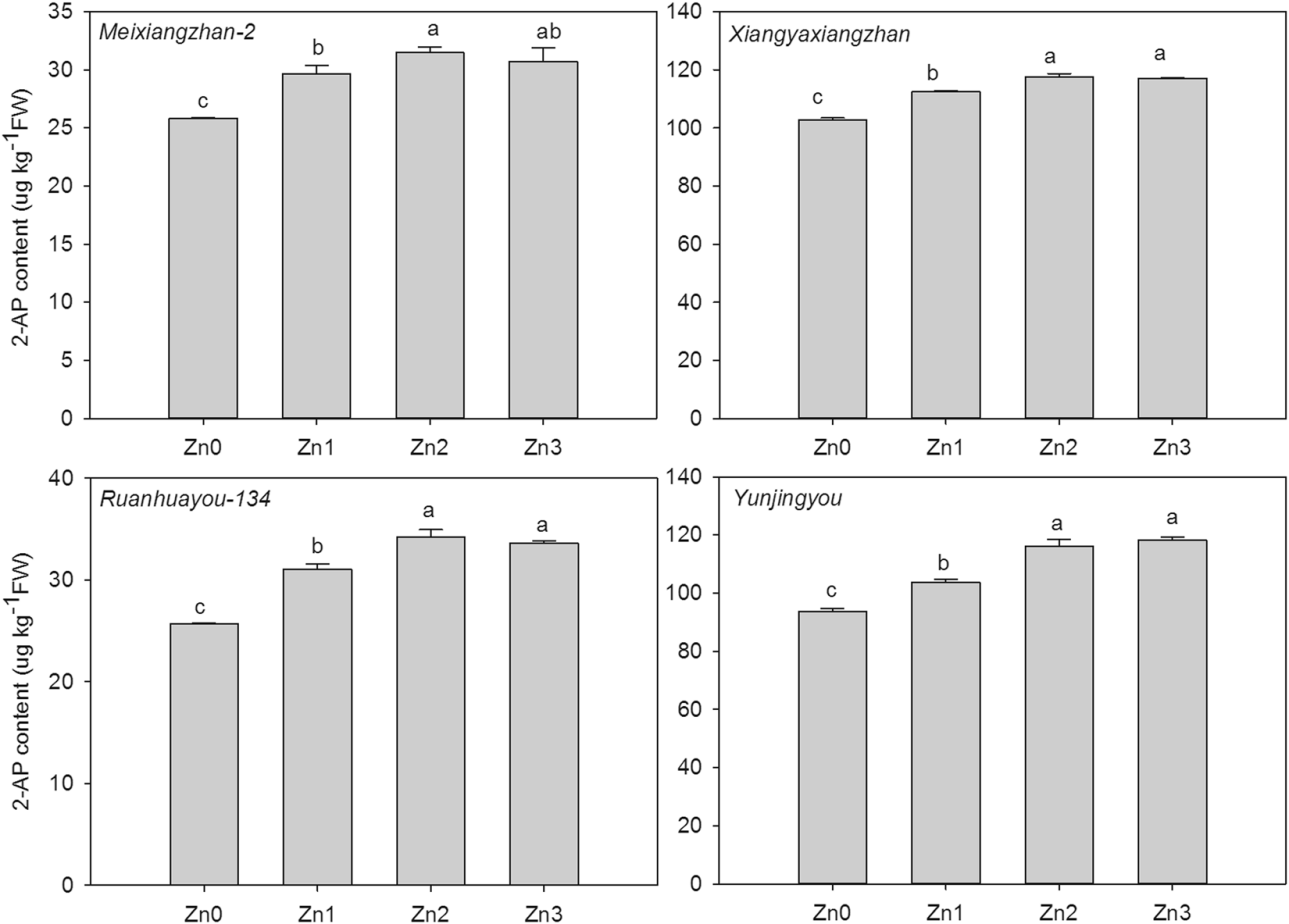
Effect of exogenous Zn on 2-AP content mature grains. Means sharing a common letter don’t differ significantly at (P ≤ 0.05) according to least significant difference (LSD) test.

As shown in Fig. 1, different Zn applications affected the 2-AP content in grains differently. For *Meixiangzhan-2*, 14.83%, 21.93% and 19.03% higher 2-AP concentrations were recorded in Zn1, Zn2 and Zn3, respectively, than in CK. For *Xiangyaxiangzhan*, compared to CK, Zn1, Zn2 and Zn3 increased the 2-AP content by 9.47%, 14.46% and 14.02%, respectively. For *Ruanhuayou-134*, the 2-AP contents in grain under the Zn1, Zn2 and Zn3 treatments were 0.20-, 0.33- and 0.30-fold higher than those under CK. For *Yunjingyou*, compared to CK, Zn1, Zn2 and Zn3 increased the 2-AP content by 10.77%, 24.07% and 26.43%, respectively.

### Enzymes involved in 2-AP biosynthesis in grains

As shown in Table 1, the application of Zn significantly improved the activities of some enzymes involved in 2-AP biosynthesis in grains. Compared with CK, the Zn1, Zn2 and Zn3 treatments significantly enhanced the activities of proline dehydrogenase (PDH), △1-pyrroline-5-carboxylic acid synthetase (P5CS) and diamine oxidase (DAO), and the enzyme activity trends were recorded as CK < Zn1 < Zn2 = Zn3. However, there was no significant difference among all treatments in ornithine aminotransferase (OAT) activity, and a similar result was also observed for betaine aldehyde dehydrogenase (BADH) activity. The enzyme activity trends were similar among the four fragrant rice genotypes.

**Table 1:** Effect of exogenous Zn application on the activities of enzymes involved in 2-AP biosynthesis in grains

Variety	Treatment	OAT (μmol g^−1^ FW)	PDH (U L^−1^ FW)	P5CS (μmol g^−1^ FW)	DAO (μmol g^−1^ FW)	BADH (U L^−1^ FW)
*Meixiangzhan-2*						
	CK	19.13a	29.22c	30.83c	0.65c	31.38a
	Zn1	19.79a	37.60b	40.14b	1.09b	31.36a
	Zn2	19.81a	43.61a	49.91a	1.29a	31.07a
	Zn3	19.99a	43.99a	51.25a	1.25a	30.80a
	Mean	19.68	38.60	43.03	1.07	31.15
*Xiangyaxiangzhan*						
	CK	22.93a	53.86c	30.52c	1.07c	31.06a
	Zn1	22.59a	61.86b	42.42b	1.57b	30.70a
	Zn2	23.07a	71.19a	62.26a	1.74a	30.92a
	Zn3	22.94a	70.79a	61.91a	1.68a	30.96a
	Mean	22.88	64.43	49.28	1.52	30.91
*Ruanhuayou-134*						
	CK	13.73a	23.53c	28.20c	0.66c	30.58a
	Zn1	13.90a	35.70b	38.35b	1.14b	31.51a
	Zn2	15.02a	46.09a	51.39a	1.28a	31.09a
	Zn3	14.01a	46.13a	52.38a	1.32a	30.88a
	Mean	14.17	37.86	42.58	1.10	31.01
*Yunjingyou*						
	CK	18.98a	49.98c	28.77c	1.03c	31.20a
	Zn1	20.94a	54.84b	42.18b	1.54b	30.32a
	Zn2	19.05a	65.12a	59.48a	1.73a	30.85a
	Zn3	18.97a	65.92a	62.01a	1.69a	30.28a
	Mean	19.49	58.96	48.11	1.50	30.66

Values sharing a common lower case letter within a column for the same variety do not differ significantly at P <0.05 according to the LSD test. Means of the two rice cultivars followed by asterisk(s) (*, **) for the growth, yield and related traits differ significantly at P <0.05 and P <0.01, respectively, according to the LSD test. The same applies below.

### Contents of precursors and related compounds involved in 2-AP biosynthesis in grains

As shown in Table 2, exogenous application of Zn regulated the contents of some compounds involved in 2-AP biosynthesis. Compared with CK, Zn application significantly increased the contents of methylglyoxal, P5C, proline and Δ1-pyrroline. The trends of methylglyoxal, P5C, proline and Δ1-pyrroline were recorded as CK < Zn1 < Zn2 = Zn3 in all fragrant rice genotypes. There were no significant differences in the GABA content among all treatments in both *Xiangyaxiangzhan* and *Yunjingyou*. However, for *Meixiangzhan-2* and *Ruanhuayou-134*, the GABA content of Zn3-treated plants was significantly higher than that of CK-treated plants, while there was no notable difference among the CK, Zn1 and Zn2 treatment groups.

**Table 2:** Effect of exogenous Zn application on precursors and related compounds involved in 2-AP biosynthesis.

Variety	Treatment	Methylglyoxal (μmol g^−1^ FW)	P5C (μmol g^−1^ FW)	GABA (μg g^−1^ FW)	Proline (ug g^−1^ FW)	Δ1-Pyrroline (mmol g^−1^ h^−1^ FW)
*Meixiangzhan-2*						
	CK	14.52c	0.82c	17.58b	32.23c	2.38c
	Zn1	15.59b	0.97b	18.01ab	42.06b	2.50b
	Zn2	16.33a	1.04a	18.66ab	51.64a	2.87a
	Zn3	16.49a	1.05a	19.03a	52.21a	2.84a
	Mean	15.73	0.97	18.32	44.53	2.65
*Xiangyaxiangzhan*						
	CK	12.37c	0.87c	16.44a	39.29c	2.15c
	Zn1	14.30b	1.02b	16.40a	54.04b	3.05b
	Zn2	16.18a	1.14a	16.65a	66.58a	3.59a
	Zn3	16.08a	1.14a	16.35a	66.43a	3.52a
	Mean	14.73	1.04	16.46	56.59	3.08
*Ruanhuayou-134*						
	CK	14.10c	0.67c	19.46b	31.53c	2.22c
	Zn1	15.56b	0.98b	19.84ab	42.32b	2.56b
	Zn2	16.52a	1.03a	20.35ab	51.28a	2.90a
	Zn3	16.50a	1.03a	20.65a	51.55a	2.75a
	Mean	15.67	0.93	20.07	44.17	2.6
*Yunjingyou*						
	CK	12.33c	0.88c	15.65a	36.42c	2.16c
	Zn1	14.51b	1.06b	15.62a	50.94b	2.78b
	Zn2	16.15a	1.17a	15.41a	60.09a	3.27a
	Zn3	16.08a	1.17a	15.66a	59.07a	3.26a
	Mean	14.77	1.07	15.58	51.63	2.87

### Net photosynthetic rate at filling stage

**Figure 2:**
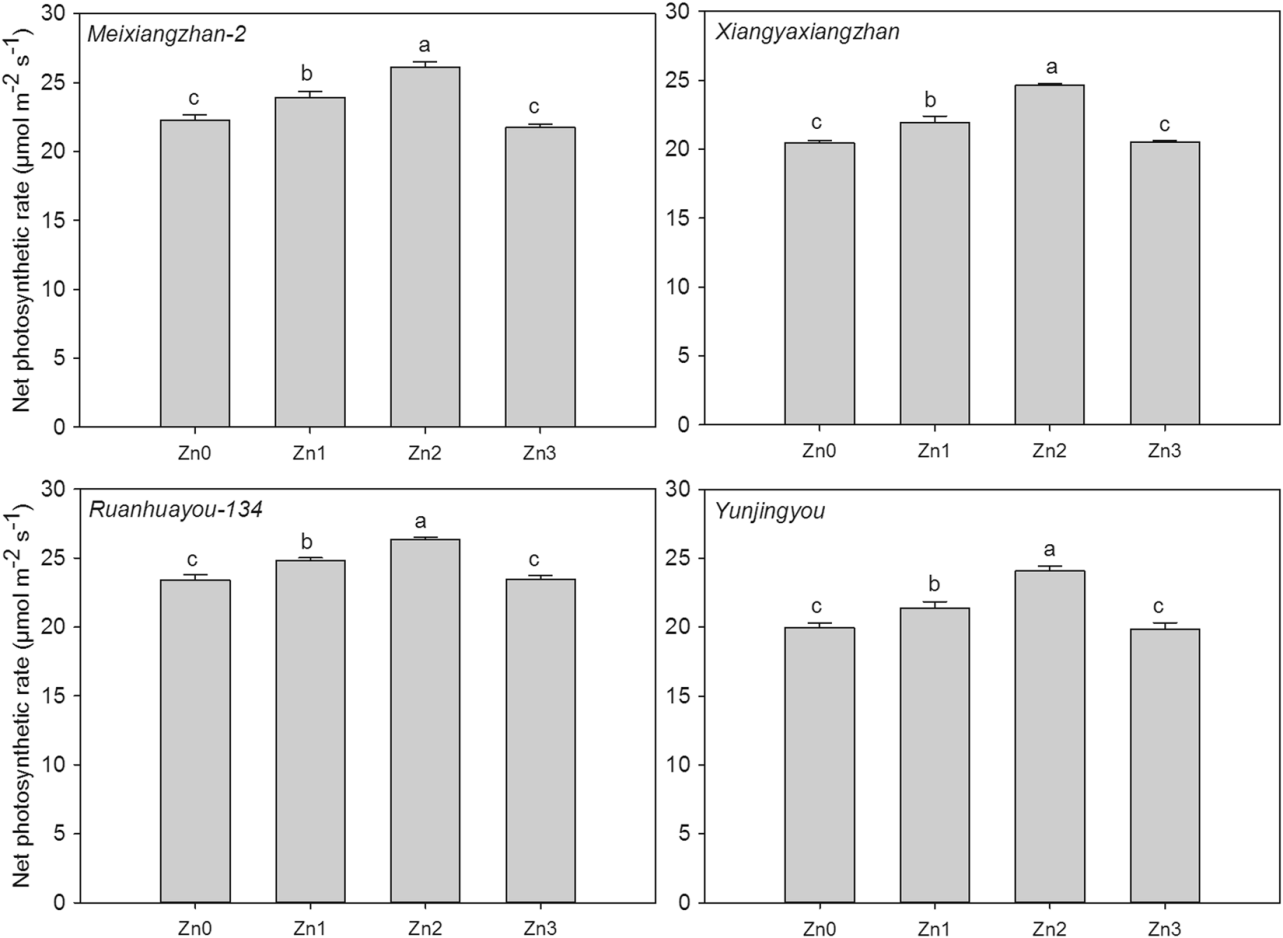
Effect of exogenous Zn on the net photosynthetic rate of fragrant rice at the filling stage. Means sharing a common letter don’t differ significantly at (P ≤ 0.05) according to least significant difference (LSD) test.

As shown in Fig. 2, the different concentrations of Zn affected the fragrant rice differently. For *Meixiangzhan-2*, 7.43% and 17.16% higher net photosynthetic rates were recorded under Zn1 and Zn2, respectively, than under CK. For *Xiangyaxiangzhan*, compared to CK, Zn1 and Zn2 significantly increased the net photosynthetic rate by 7.26% and 20.28%, respectively. For *Ruanhuayou-134*, the net photosynthetic rates under Zn1 and Zn2 were 0.06 and 0.13-fold higher, respectively, than those under CK. For *Yunjingyou*, compared to CK, Zn1 and Zn2 increased the net photosynthetic rate at the filling stage by 7.14% and 20.57%, respectively.

### Zn content in grains

**Figure 3:**
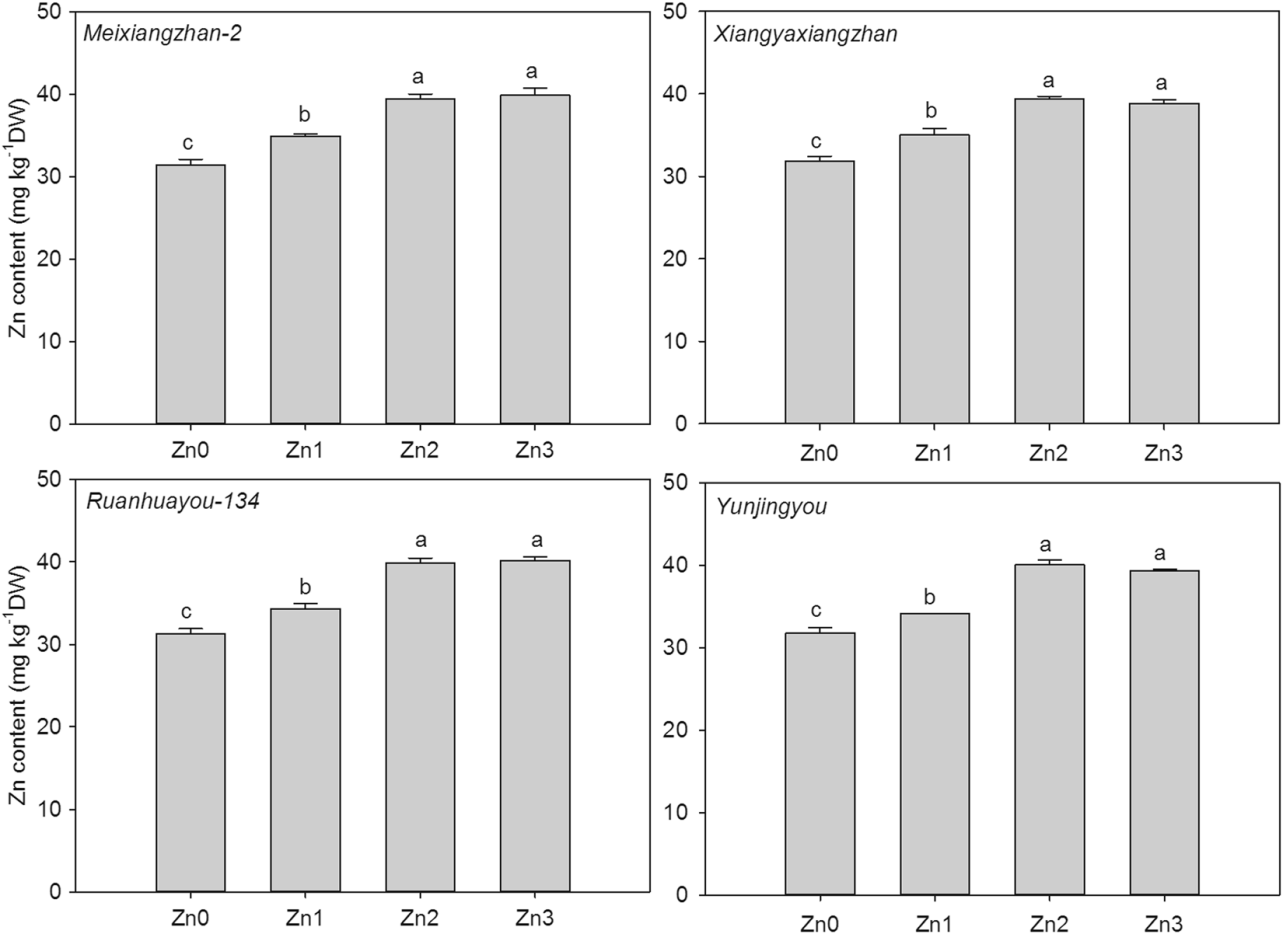
Effect of exogenous Zn on the Zn content of mature grains. Means sharing a common letter don’t differ significantly at (P ≤ 0.05) according to least significant difference (LSD) test.

As shown in Fig. 3, exogenous Zn application significantly improved the Zn content in the grains of fragrant rice. For *Meixiangzhan-2*, 11.16%, 25.53% and 26.78% higher Zn concentrations were recorded under Zn1, Zn2 and Zn3, respectively, than under CK. For *Xiangyaxiangzhan*, compared to CK, Zn1, Zn2 and Zn3 increased the 2-AP content by 10.10%, 23.81% and 22.08%, respectively. For *Ruanhuayou-134*, the 2-AP contents in grain under Zn1, Zn2 and Zn3 were 0.10-, 0.27- and 0.28-fold higher, respectively, than those under CK. For *Yunjingyou*, compared to CK, Zn1, Zn2 and Zn3 increased the 2-AP content by 7.33%, 26.04% and 23.77%, respectively.

### Grain yield and yield-related trials

As shown in Table 3, Zn application modulated fragrant rice yield formation to a certain degree. Compared with CK, all Zn treatments (except Zn3 in *Meixiangzhan-2* and *Ruanhuayou-134*) significantly increased the grain yield, and the highest yield was recorded with the Zn2 treatment for all rice genotypes. Furthermore, the Zn2 treatment also significantly improved the seed-setting rate and 1000-grain weight for all rice varieties. There was no notable difference among the effects of CK, Zn1, Zn2 and Zn3 in both the effective panicle number and grain number.

**Table 3:** Effect of exogenous Zn application on fragrant rice yield and yield-related trials.

Variety	Treatment	Effective panicle number per m^2^	Grain number per panicle	Seed-setting rate (%)	1000-grain weight (g)	Yield (t ha^−1^)
*Meixiangzhan-2*						
	CK	328.00a	161.82a	78.72b	20.12b	4.92c
	Zn1	320.00a	162.30a	80.23ab	20.34ab	5.25b
	Zn2	330.66a	161.51a	81.89a	20.60a	5.40a
	Zn3	327.66a	162.09a	80.28ab	20.19ab	5.16bc
	Mean	326.58	161.93	80.28	20.32	5.21
*Xiangyaxiangzhan*						
	CK	303.66a	126.52a	78.78c	18.78b	4.58c
	Zn1	297.33a	127.51a	81.18b	19.02ab	4.83b
	Zn2	301.33a	127.26a	82.84a	19.12a	5.02a
	Zn3	305.66a	127.92a	78.78c	18.79b	4.75b
	Mean	302.00	127.30	80.40	18.93	4.90
*Ruanhuayou-134*						
	CK	282.33a	266.36a	77.85c	17.45b	4.75c
	Zn1	278.33a	269.16a	79.48b	17.55b	4.92b
	Zn2	276.66a	266.89a	82.48a	17.86a	5.13a
	Zn3	270.33a	267.60a	79.32b	17.40b	4.83bc
	Mean	276.91	267.50	79.79	17.57	4.96
*Yunjingyou*						
	CK	287.66a	111.17a	82.00c	24.08b	4.08c
	Zn1	287.00a	113.17a	84.60b	24.32ab	4.21b
	Zn2	291.00a	112.60a	87.31a	24.56a	4.47a
	Zn3	281.67a	112.69a	83.62b	24.14b	4.19b
	Mean	286.83	112.41	84.38	24.27	4.31

### Correlation analysis

As shown in Table 4, the PDH, P5CS and DAO activities were all significantly and positively correlated with the 2-AP content in mature grains. Similar correlations were also observed between the 2-AP content and the contents of methylglyoxal, P5C, proline, and Δ1-pyrroline.

**Table 4:** Correlation between 2-AP content and enzymes and compounds involved in 2-AP biosynthesis in different fragrant rice genotypes.

	*Meixiangzhan-2*	*Xiangyaxiangzhan*	*Ruanhuayou-134*	*Yunjingyou*
OAT activity	0.9370	0.1804	0.6886	−0.2349
PDH activity	0.9772*	0.9787*	0.9910**	0.9934**
P5CS activity	0.9492*	0.9562*	0.9688*	0.9999**
DAO activity	0.9972**	0.9917**	0.9854*	0.9328*
BADH activity	−0.6758	−0.3650	0.4894	−0.5663
Methylglyoxal content	0.9670*	0.9872**	0.9973**	0.9839*
P5C content	0.9875*	0.9896*	0.9700*	0.9786*
GABA content	0.8628	0.2125	0.9155	−0.4350
Proline content	0.9616*	0.9914**	0.9906**	0.9719*
Δ1-Pyrroline content	0.8879*	0.9995**	0.9852*	0.9863*

^*^Significant at P <0.05; **Significant at P <0.01. The same applies below.

As shown in Table 5, there was no significant correlation between panicle number and grain yield for all rice genotypes. Similar conditions were also found between grain number and grain yield. However, both the seed-setting rate and 1000-grain weight had a significant and positive correlation with the grain yield of the four fragrant rice genotypes.

**Table 5:** Correlation between grain yield and yield-related trials in different fragrant rice genotypes.

	*Meixiangzhan-2*	*Xiangyaxiangzhan*	*Ruanhuayou-134*	*Yunjingyou*
Panicle number per m^2^	0.4939	−0.3831	−0.0010	0.4461
Grain number per panicle	−0.2517	−0.2836	0.0233	0.0484
Seed-setting rate	0.7114**	0.6140*	0.8685**	0.7495**
1000-grain weight	0.6795*	0.5675*	0.6801*	0.6569*

## Discussion

**Figure 4:**
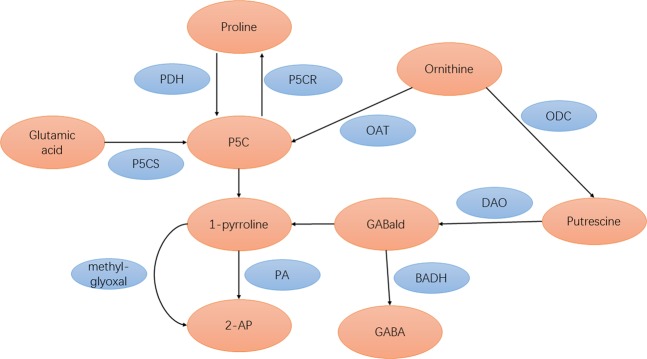
Possible mechanism of 2-AP formation in fragrant rice.

2-AP biosynthesis in fragrant rice is a complicated phenomenon. Many researchers have conducted studies on this process. For example, Yoshihashi^12^ showed that 2-AP was not produced at all by the cooking or postharvest processes according to a stable isotope dilution method. Moreover, Yoshihashi also discovered that adding proline, ornithine and glutamic acid to the solution increased the content of 2-AP in fragrant rice seedlings and calli^13^. In 2008, Chen^14^ demonstrated that the presence of a dominant Badh2 allele encoding betaine aldehyde dehydrogenase (BADH) inhibits the biosynthesis of 2-AP in rice because BADH decreased 2-AP formation by catalysing 4-aminobutyraldehyde (GABald) into GABA, while GABald may be one of the precursors of 2-AP biosynthesis. The investigation of Mo^15^ also indicated that there was a correlation between the GABA content and the 2-AP content in fragrant rice grains. Furthermore, the study of Poonlaphdecha^16^ proved that 1-pyrroline is an important precursor in the biosynthesis of 2-AP in fragrant rice by using rice callus cultures. In recent years, it has generally been believed that five enzymes play a key role in the process of 2-AP biosynthesis in fragrant rice: PDH, P5CS, OAT, DAO and BADH^17^. Therefore, one possible 2-AP synthesis pathway in fragrant rice is shown in Fig. 4.

**Figure 5:**
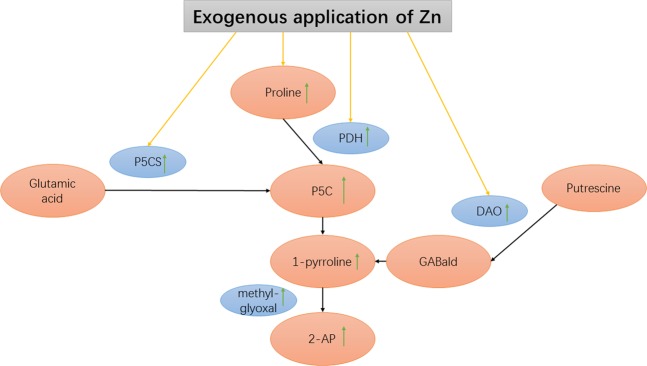
Regulation of 2-AP biosynthesis by Zn application in fragrant rice.

The present study revealed the modulation of 2-AP biosynthesis and yield formation by Zn in different fragrant rice genotypes. The results showed that three concentrations of Zn all improved the 2-AP content in mature grains, and the highest improvement was recorded with Zn2 for all fragrant rice genotypes. The increase in 2-AP due to Zn application could be attributed to the enhanced activities of enzymes, including PDH, P5CS and DAO. This study showed that exogenous Zn application at the heading stage promoted the activities of PDH and P5CS during the filling stage, and the activities of these enzymes all had a significant positive correlation with the 2-AP content, which was corroborated by the early research of Deng^18^. PDH is a key enzyme in mitochondria that catalyses the degradation of proline to P5C, while regulatory feedback of P5CS plays an important role in controlling the plant proline level because P5CS has glutamyl-gamma-semialdehyde deaminase (GSADH) and glutamate kinase (gamma-GK) activities, and it can catalyse the phosphorylation of glutamate to glutamic semialdehyde (GSA) and reduce GSA^19^. Li (Li *et al*.^4^) indicated that the 2-AP concentration in fragrant rice grains was positively correlated with the activities of PDH and P5CS, along with the contents of proline, P5C, methylglyoxal and △1-pyrroline in fragrant rice, which revealed that a higher 2-AP concentration is related to increased activities of the enzymes involved in its biosynthesis. Higher contents of proline, P5C, methylglyoxal and △1-pyrroline, which were accepted as precursors and intermediates for 2-AP biosynthesis, also appeared in the present study following the Zn treatments. On the other hand, DAO transforms putrescine into GABald, which further cyclizes spontaneously to Δ1-pyrroline, or synthesizes GABA, depending on the absence or presence of functional BADH_2_ enzyme^20^. Considering the changes in enzyme activities and contents of precursors and intermediates, the modulation of 2-AP biosynthesis by exogenous Zn application in fragrant rice may occur by the mechanisms shown in Fig. 5.

In addition, we observed that exogenous application of Zn at a 1.00 g L^−1^ concentration greatly increased the grain yield for the four fragrant rice genotypes. This increase could be explained by the increase in the seed-setting rate and 1000-grain weight. The increase may further be attributed to the improvement in the net photosynthetic rate during the grain filling stage due to exogenous Zn application at the heading stage. Photosynthesis is one of the most important physiological processes in rice and significantly influences the grain yield^21^. An early study illustrated the importance of photosynthesis during the grain filling phase to rice yield by showing that higher net photosynthesis at the filling stage could increase grain yield^22^. Our result was consistent with those of Tavallali^23^, who demonstrated that the application of Zn could increase the net photosynthetic rate in pistachio. Previous studies also revealed that carbonic anhydrase (CA), a zinc-containing metalloenzyme that is widely distributed in plants and mainly exists in chloroplasts, could catalyse the hydration of carbon dioxide, promote the fixation of carbon dioxide in photosynthesis, and reduce the activity of CA due to zinc deficiency^24,25^.

## Conclusion

In conclusion, foliar application of Zn at the heading stage may have a great effect on 2-AP biosynthesis in fragrant rice. Among the studied fragrant rice varieties, it was shown in *Meixiangzhan-2*, *Xiangyaxiangzhan*, *Ruanhuayou-134* and *Yunjingyou* that exogenous application of Zn regulated the enzymes involved in 2-AP biosynthesis, including PDH, P5CS and DAO, and increased the contents of precursors, such as proline and P5C. Moreover, Zn application upregulated the net photosynthetic rate during the grain filling stage and increased the grain yield of fragrant rice. The Zn content in grains also increased due to foliar application of Zn. Considering that the highest yield and 2-AP content were recorded with the Zn2 treatment for all fragrant rice genotypes, 1.00 g L^−1^ can be recommended as the best foliar application concentration. To delve more deeply into the mechanism of 2-AP formation under the influence of Zn application, further work should be done at the molecular level.

## Materials and Methods

### Plant materials and growing conditions

**Figure 6:**
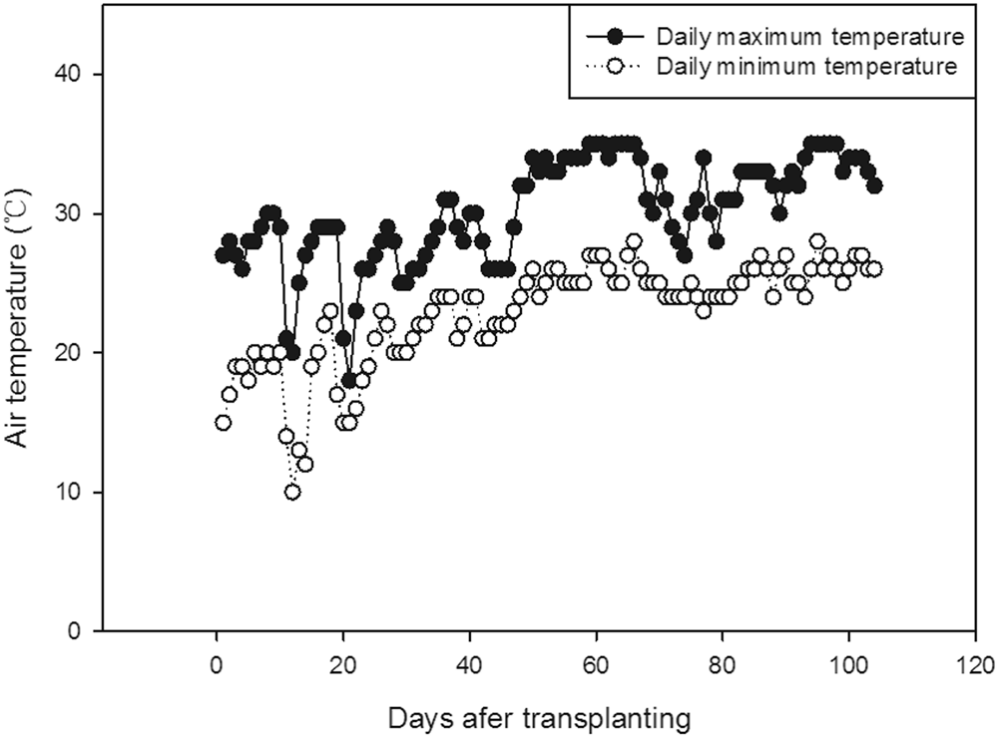
Daily maximum and minimum temperatures during the experiment

Seeds of four fragrant rice varieties, ‘*Meixiangzhan-2*’, ‘*Xiangyaxiangzhan*’, ‘*Ruanhuayou-134*’, and ‘*Yunjingyou*’, which are widely grown fragrant rice varieties in Guangdong Province, China, were provided by the College of Agriculture, South China Agricultural University, Guangzhou China, and used in the present study. A field experiment during 2018 was conducted at the Experimental Research Farm, College of Agriculture, South China Agricultural University, Zengcheng (23°16′N, 113°22′E and 11 m above mean sea level), China. The experimental soil was sandy loam consisting of 15.91 g/kg organic matter, 1.34 g/kg total nitrogen, 55.16 mg/kg available nitrogen, 1.40 g/kg total phosphorus, 18.11 mg/kg available phosphorus, 13.22 mg/kg total potassium, and 128.06 mg/kg available potassium, with a soil pH of 6.70. The experimental site has a subtropical monsoon climate. The temperatures during the experiment are shown in Fig. 6. Before sowing, the seeds were soaked in water for 24 h, germinated in manually climate-controlled boxes for another 12 h, and then shade-dried, followed by sowing in polyvinyl chloride trays for nursery cultivation. Then, 20-day-old seedlings were transplanted to fields at a planting distance of 30 × 12 cm^2^.

### Treatments and plant sampling

The four Zn treatments were set as follows: overhead sprinkling with 0, 0.50, 1.00 and 2.00 g·L^−1^ zinc chloride at the heading stage; these treatments were named CK, Zn1, Zn2 and Zn3, respectively. The treatments were arranged in a randomized complete block design (RCBD) in triplicate with a net plot size of 16 m^2^ (4 m·4 m). A total of 1.20 litres of the corresponding liquid was applied to each plot. Fresh grains were separated and collected from the rice plants after 15 days at the heading stage, washed with double-distilled water and stored at −80 °C for physio-biochemical analysis. At maturity, fresh grains were separated and collected from the rice plants and stored at −80 °C for 2-AP determination.

### Determination of the 2-acetyl-1-pyrroline (2-AP) content in grains

Approximately 0.5 g of fresh grains were homogenized in 5 mL of 60% ethanol and treated for 4 h in an oscillator (HZS-H, China) at 200 oscillations per minute. The absorbance after the reaction was read at 645 nm, and the 2-AP amount was expressed as µg g^**−**1^. The 2-AP concentration was determined by the synchronization distillation and extraction (SDE) method combined with a GCMS-QP 2010 Plus (Shimadzu Corporation, Japan) according to Mo^26^, and the 2-AP contents were expressed as ug kg^**−**1^.

### Determination of the Δ1-pyrroline, proline, pyrroline-5-carboxylic acid (P5C), γ-aminobutyric acid (GABA) and methylglyoxal contents in grains

The Δ1-pyrroline content in grains was estimated by the method of Hill^27^. The amount of △^1^-pyrroline in reaction mixtures containing 1, 4-diaminobutane was determined immediately and after 30 min at room temperature. Proline contents in grains were estimated according to the method of Bates^28^ using ninhydrin, and the absorbance was read at 520 nm. The P5C concentration was estimated following the method of Wu^29^. The mixture contained 0.2 ml of enzyme extraction supernatant, 0.5 ml of 10% trichloroacetic acid (TCA) and 0.2 ml of 40 mM 2-aminobenzaldehyde. After the reaction, the absorbance was read at 440 nm. The GABA content was measured according to the methods described by Zhao^30^. Grains of approximately 0.5 g were homogenized in 5 ml of 60% ethanol and treated for 4 h in an oscillator (HZS-H, China) at 200 oscillations per minute. The absorbance after the reaction was read at 645 nm. The content of methylglyoxal was estimated by the methods described by Banu^31^. The reaction system was 1 ml, with 250 L 7.2 mM 1, 2-diaminobenzene, 100 L 5 M perchloric acid, and 650 μL extract. The absorbance was determined at 336 nm.

### Determination of proline dehydrogenase (PDH), △1-pyrroline-5-carboxylic acid synthetase (P5CS), ornithine aminotransferase (OAT), diamine oxidase (DAO) and betaine aldehyde dehydrogenase (BADH) activities in grains

The PDH activity was assayed by following the methods of Ncube^32^. Following the reaction, the absorbance was read at 440 nm, and the activity was calculated using a molar extinction coefficient. The activity of P5CS was estimated according to the methods described by Zhang^33^. The reaction mixture included 50 mM Tris-HCL buffer, 20.0 mM MgCl2, 50 mM sodium glutamate, 10 mM ATP, 100 mM hydroxamate-HCL and 0.5 ml of enzyme extract. OAT activity was measured according to the methods of Chen^34^. The absorbance of the supernatant fraction was read at 440 nm, and the activity was calculated by the extinction coefficient 2.68 mM^**−1**^ cm^**−**1^. The DAO activity was assayed using the methods described by Su^35^. Reaction solutions (2.9 mL) contained 2.0 mL of 70 mmol/L sodium phosphate buffer (pH 6.5), 0.5 mL of crude enzyme extracts, 0.1 mL of horseradish peroxidase (250 U/mL), and 0.2 mL of 4-aminoantipyrine/N,N-dimethylaniline. The betaine aldehyde dehydrogenase 2 (BADH2) enzyme activity was measured using a Plant PRODH ELISA Kit (Mlbio, Shanghai, China) according to the manufacturer’s protocol. The absorbance was measured using a BioTek Epoch spectrophotometer (BioTek, Vermont, USA) at 450 nm.

### Determination of photosynthesis

At 15 days after the heading stage, a portable photosynthesis system (LI-6400, LI-COR, USA) was used to determine the net photosynthetic rate from 09:00–10:30 a.m. with the following adjustments: photosynthetically active radiation at the leaf surface was 1100–1200 μmol m^−2^ s^−1^, the ambient CO_2_ concentration was 385.0–400.0 μmol mol^−1^, and the air temperature was 31.5 ± 0.5 °C with 60–80% RH.

### Determination of yield and yield-related traits

At the maturity stage, the rice grains were harvested from the five-unit sampling areas (1.75 m^2^) in each plot and then threshed by machine. The harvested grains were sun-dried and weighed to determine the grain yield. Twenty hills of rice from different locations in each plot were sampled to estimate the average effective panicle number per hill. Then, six representative hills of plants were taken to estimate the yield-related traits.

### Determination of Zn content in grains

Approximately 2.000 g of the crushed sample was weighed and placed into a digestive tube with a concentrated nitric acid and perchloric acid mixture in a 4:1 ratio. The mixture was shaken well and left in a fume hood for cold digestion overnight. The next day, the sample was heated and digested in a digestive furnace. The procedure of heat digestion was as follows: 60 min digestion at 60°C, 60 min digestion at 100°C, 100 min digestion at 180°C, 180 min digestion at 195°C. If the liquid became brown-black during digestion, a small amount of nitric acid was added after cooling, and then the digestive liquid was heated until it was colourless or slightly transparent after cooling. The digestive tube was washed with ultra-pure water and filtered through medium-speed qualitative filter paper. The volume of the liquid was set to 25 ml, and the blank was made at the same time. The filtrate was determined by atomic absorption spectrophotometry, and then the zinc content in the sample was calculated based on a standard curve.

### Statistical analysis

Data were analysed with Statistix 8.1 (Analytical Software, Tallahassee, FL, USA), while differences among means were separated by using the least significant difference (LSD) test at the 5% probability level. Graphs were drawn with Sigma Plot 14.0 (Systat Software Inc., California, USA).
